# 782 Process for Estimating Burn Clinical Research Staffing Needs and Measuring Work Output and Productivity

**DOI:** 10.1093/jbcr/irae036.323

**Published:** 2024-04-17

**Authors:** Joan Wilson, Austin Price, Zaheed Hassan, Bounthavy F Homsombath, Shawn P Fagan, Kade Hardy, Rajiv Sood

**Affiliations:** JMS Research Foundation, Inc., Augusta, GA; JMS Burn Center at Doctors Hospital Augusta, Augusta, GA; JMS Burn Center at Doctors Hospital, Augusta, GA; Joseph M. Still Research Foundation, Inc., Augusta, GA; JMS Research Foundation, Inc., Augusta, GA; JMS Burn Center at Doctors Hospital Augusta, Augusta, GA; JMS Burn Center at Doctors Hospital, Augusta, GA; Joseph M. Still Research Foundation, Inc., Augusta, GA; JMS Research Foundation, Inc., Augusta, GA; JMS Burn Center at Doctors Hospital Augusta, Augusta, GA; JMS Burn Center at Doctors Hospital, Augusta, GA; Joseph M. Still Research Foundation, Inc., Augusta, GA; JMS Research Foundation, Inc., Augusta, GA; JMS Burn Center at Doctors Hospital Augusta, Augusta, GA; JMS Burn Center at Doctors Hospital, Augusta, GA; Joseph M. Still Research Foundation, Inc., Augusta, GA; JMS Research Foundation, Inc., Augusta, GA; JMS Burn Center at Doctors Hospital Augusta, Augusta, GA; JMS Burn Center at Doctors Hospital, Augusta, GA; Joseph M. Still Research Foundation, Inc., Augusta, GA; JMS Research Foundation, Inc., Augusta, GA; JMS Burn Center at Doctors Hospital Augusta, Augusta, GA; JMS Burn Center at Doctors Hospital, Augusta, GA; Joseph M. Still Research Foundation, Inc., Augusta, GA; JMS Research Foundation, Inc., Augusta, GA; JMS Burn Center at Doctors Hospital Augusta, Augusta, GA; JMS Burn Center at Doctors Hospital, Augusta, GA; Joseph M. Still Research Foundation, Inc., Augusta, GA

## Abstract

**Introduction:**

In the setting of burn clinical research, no widely adopted tool exists that helps predict staffing needs. Additionally, there is very little reported on burn quality metrics at all. This results in a gap in practice when one considers that there is always a mix of funded versus non-funded projects, all of which require varying levels of workload and input. Adequate staffing is required, regardless of the work mix. While a few sources are available in the oncology research setting, none offer a valid tool for determining staffing for clinical research coordinators (CRCs). A comprehensive search for such a tool identified the TOPS tool (Tool for Operational Protocol Scoring). Because the tool considers protocol complexity, it gives the user a valid means of assigning needs for any project. This tool has allowed us to justify the number of protocols we can feasibly take on considering the number of CRCs on staff and remain fiscally and administratively efficient.

**Methods:**

The TOPS tool was implemented to determine the overall complexity score for the work at hand and to verify that the number of coordinators on staff was adequate. Additionally, a complete list of tasks was developed to account for non-research-related work (i.e., admin, meeting attendances, daily rounds, training, etc.). Each employee estimated the time spent on every task they performed. To validate this, all staff were then asked to document their time. The Director and lead coordinator documented time spent on all other tasks not assigned to CRCs. At the end of 3 months, the time documented was very similar to the time estimated initially, so this time was then used to calculate workload distribution by percent effort. The total number of work hours in a year was used to calculate the portion of work time spent on each task to obtain percentage effort.

**Results:**

According to the TOPS tool, protocol complexity indicated that staff was adequate to cover staffing needs at that time. All staff members worked at over 100% effort.

**Conclusions:**

The TOPS tool is a very close predictor for staffing implementation for research work of varying complexity. Validating percent effort by role further validates staffing and work assignments. These activities are also helpful in planning future research activities and timelines.

**Applicability of Research to Practice:**

There currently is a gap in practice to identify proper tools to predict the need for staffing of a burn research office. Specifically, how many coordinators does it take to run the number of studies and projects at any given time? This tool addresses that gap.

